# Pericardial effusion in an infant with cardiac capillary hemangioma: a case report

**DOI:** 10.1093/jscr/rjaf812

**Published:** 2025-10-14

**Authors:** Alwaleed Al-Dairy, Rama Sleem, Rami Hamdan, Ghaith Hasan, Ahmad Al-Bitar

**Affiliations:** Department of Cardiac Surgery, Faculty of Medicine, Damascus University, Fayez Mansour Street, PO Box 222, Damascus, Syrian Arab Republic; Faculty of Medicine, Damascus University, Fayez Mansour Street, PO Box 222, Damascus, Syrian Arab Republic; Faculty of Medicine, Damascus University, Fayez Mansour Street, PO Box 222, Damascus, Syrian Arab Republic; Faculty of Medicine, Damascus University, Fayez Mansour Street, PO Box 222, Damascus, Syrian Arab Republic; Faculty of Medicine, Damascus University, Fayez Mansour Street, PO Box 222, Damascus, Syrian Arab Republic

**Keywords:** cardiac capillary hemangioma, infant cardiac tumours, pericardial effusion, case report

## Abstract

Primary cardiac tumours in infants are rare, mostly benign, but can cause life-threatening complications. We present a 2-month-old female with respiratory distress, poor feeding, and prior pericardial effusion/cardiac arrest. Transthoracic echocardiography revealed a right atrial mass with massive pericardial effusion. Hemodynamic instability prompted urgent surgery. Intraoperatively, the tumour infiltrated the right atrial anterior wall and atrioventricular groove; subtotal resection and atrial reconstruction using a bovine pericardial patch were performed. Histopathology confirmed benign capillary hemangioma (CD31/CD34 positive). The infant recovered well, remaining recurrence-free at one year. This case illustrates the severe potential of cardiac capillary hemangiomas in infants. Despite critical location and rapid progression, surgical debulking stabilized hemodynamics despite incomplete resection.

## Key message

Cardiac hemangiomas are rare in infants, and despite their benign nature, they can cause severe symptoms due to location and size. In unstable cases, transthoracic echocardiography is often the only feasible imaging modality. When complete resection is not possible, subtotal excision can still be effective. With timely surgery and close follow-up, outcomes are generally favourable.

## Introduction

Primary cardiac tumours (PCTs) are exceptionally rare, with ~90% being benign [1]. In adults, myxomas are the most common type, whereas rhabdomyomas account for most cases in infants and children [2]. Cardiac hemangiomas, a rarer subtype comprising ~2.8% of all PCTs [3], can arise from any cardiac structure but most commonly involve the right atrium. Histologically, they are classified as cavernous, capillary, or arteriovenous [1].

While often asymptomatic, clinical manifestations depend on the tumour’s size and location, with documented symptoms including arrhythmias, dyspnea, and heart failure [4]. Transthoracic echocardiography (TTE) is the first-line imaging modality for evaluation [5], with computed tomography (CT) and magnetic resonance imaging (MRI) used as adjuncts for surgical planning. Surgical excision is the mainstay of treatment in symptomatic cases [1]. We present the case of a 2-month-old female infant who required emergency surgery for cardiogenic shock and a massive pericardial effusion caused by a right atrial capillary hemangioma.

## Case presentation

**Figure 1:**
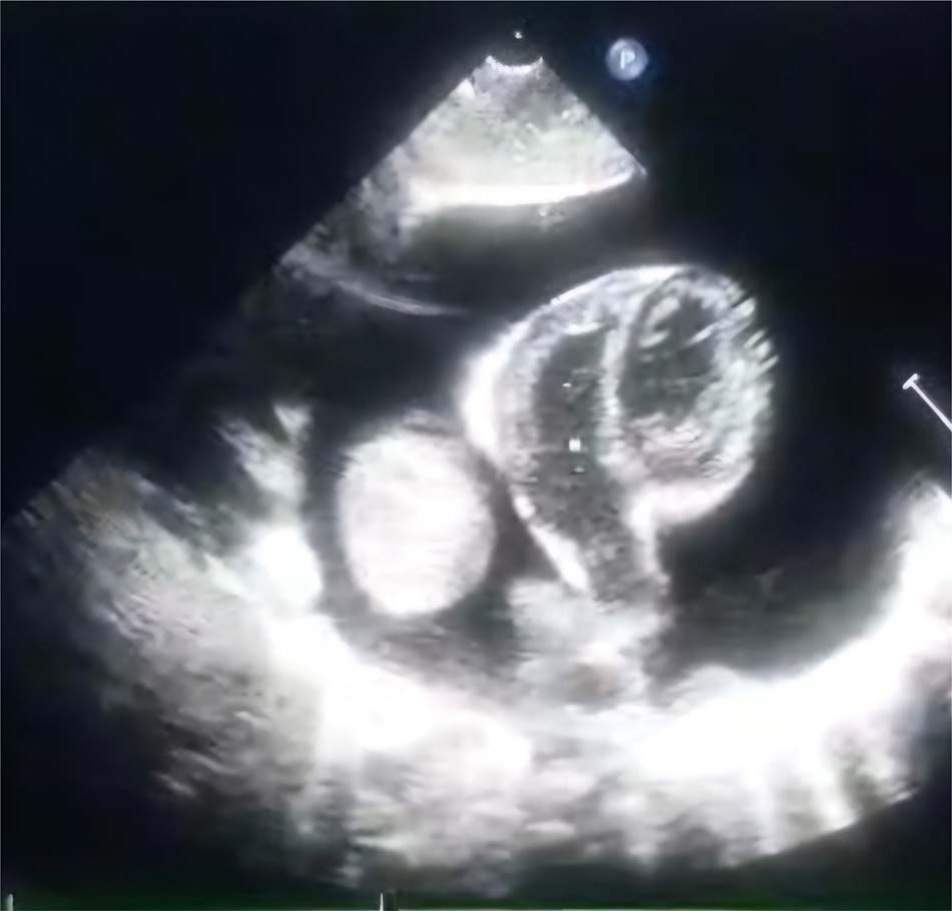
TTE view showing the massive pericardial effusion and the mass.

**Figure 2:**
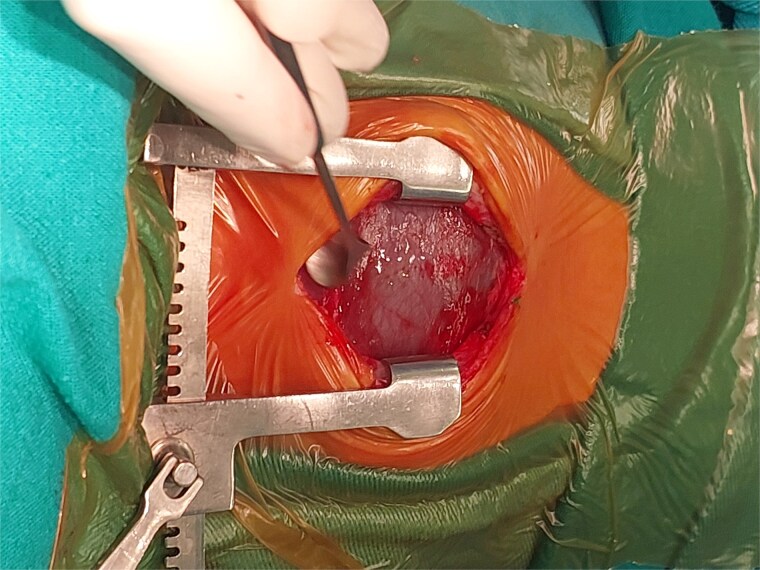
Intraoperative image showing the stretched pericardium due to massive effusion.

**Figure 3:**
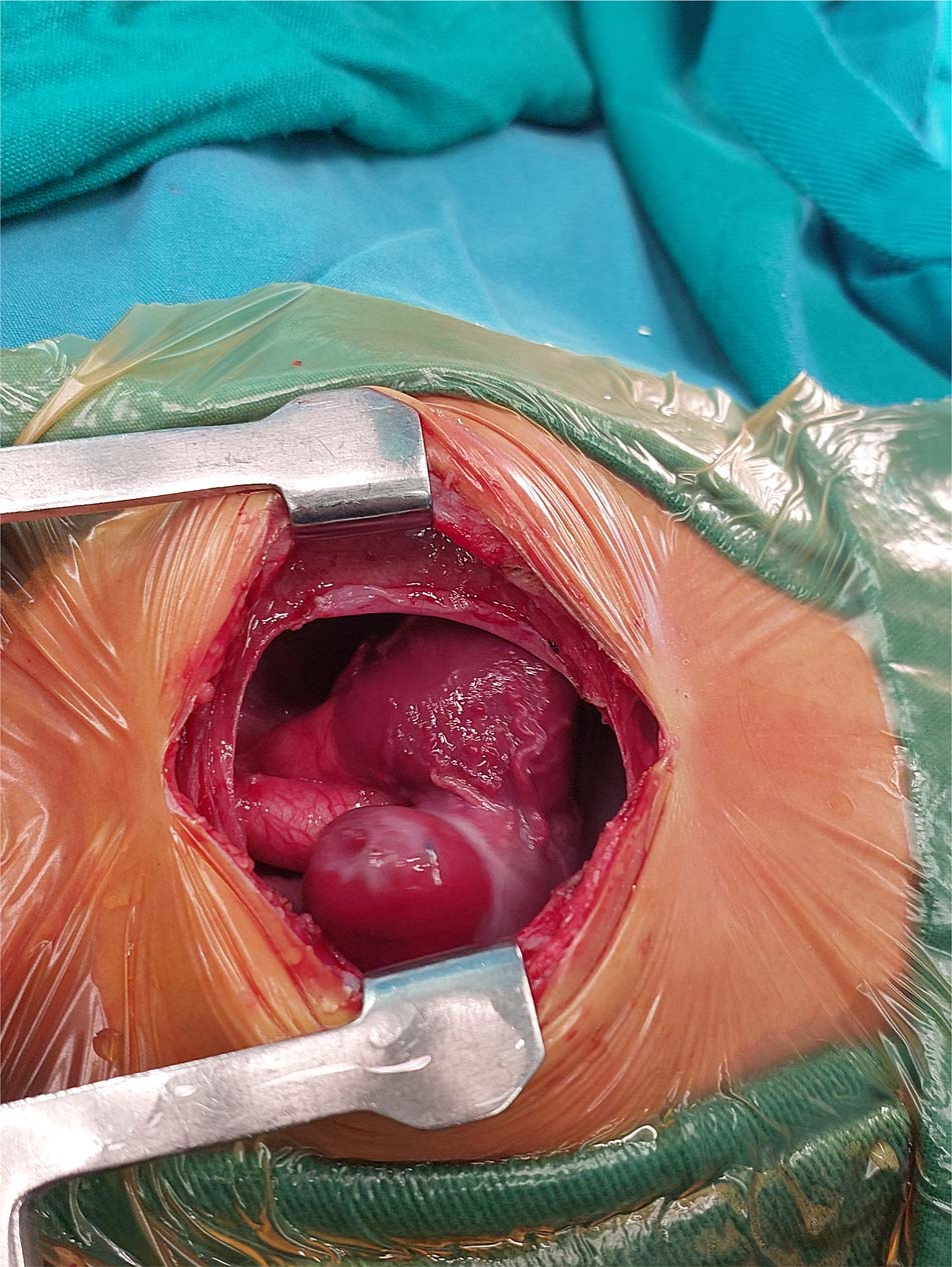
Intraoperative image showing the mass in the right atrium after opening the pericardium.

**Figure 4:**
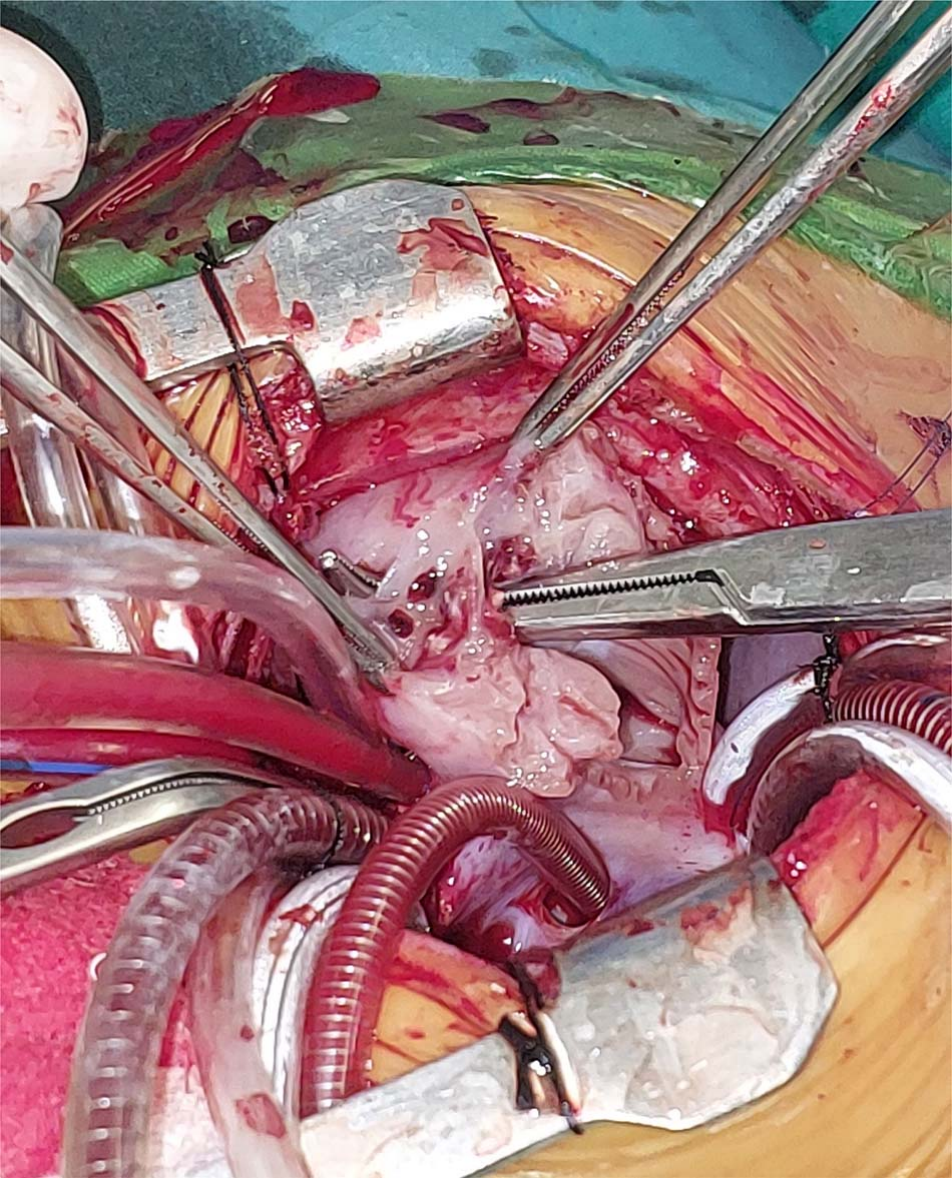
Intraoperative image showing the opened right atrium and the mass partially resected.

**Figure 5:**
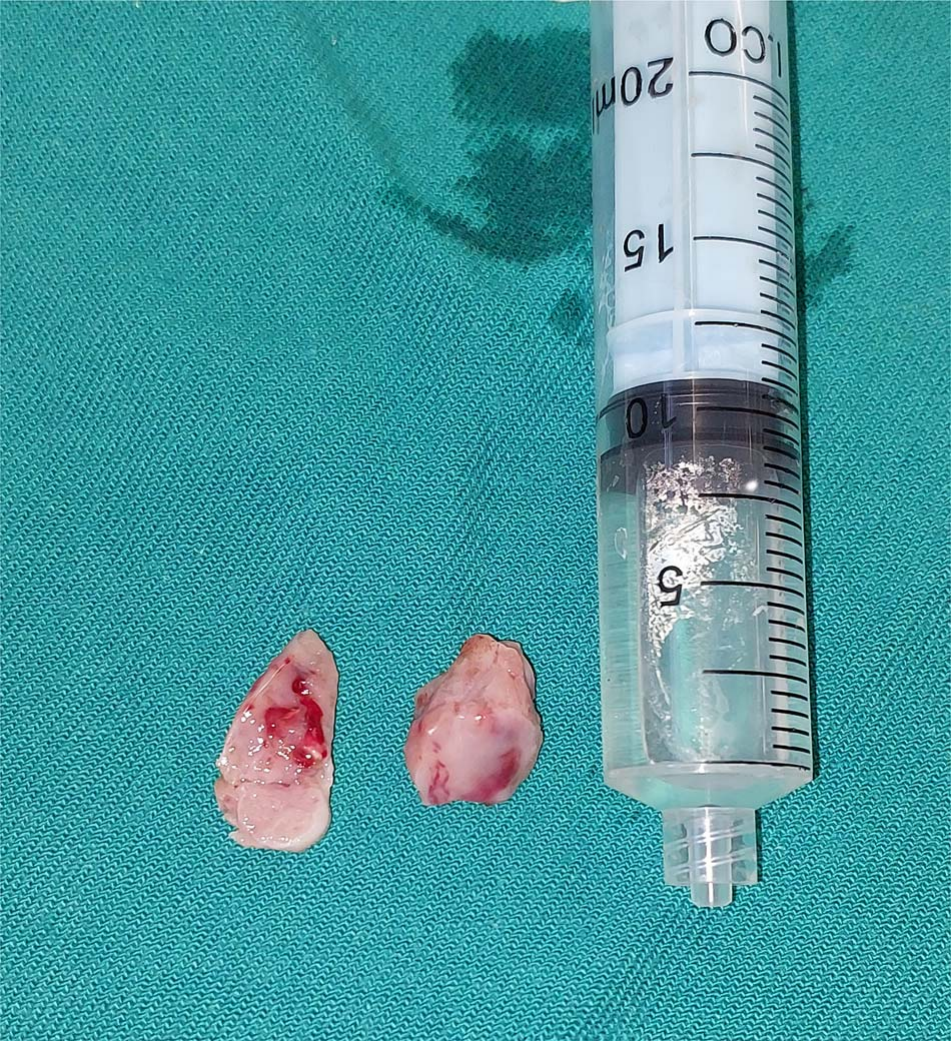
Intraoperative image showing the resected mass.

**Figure 6:**
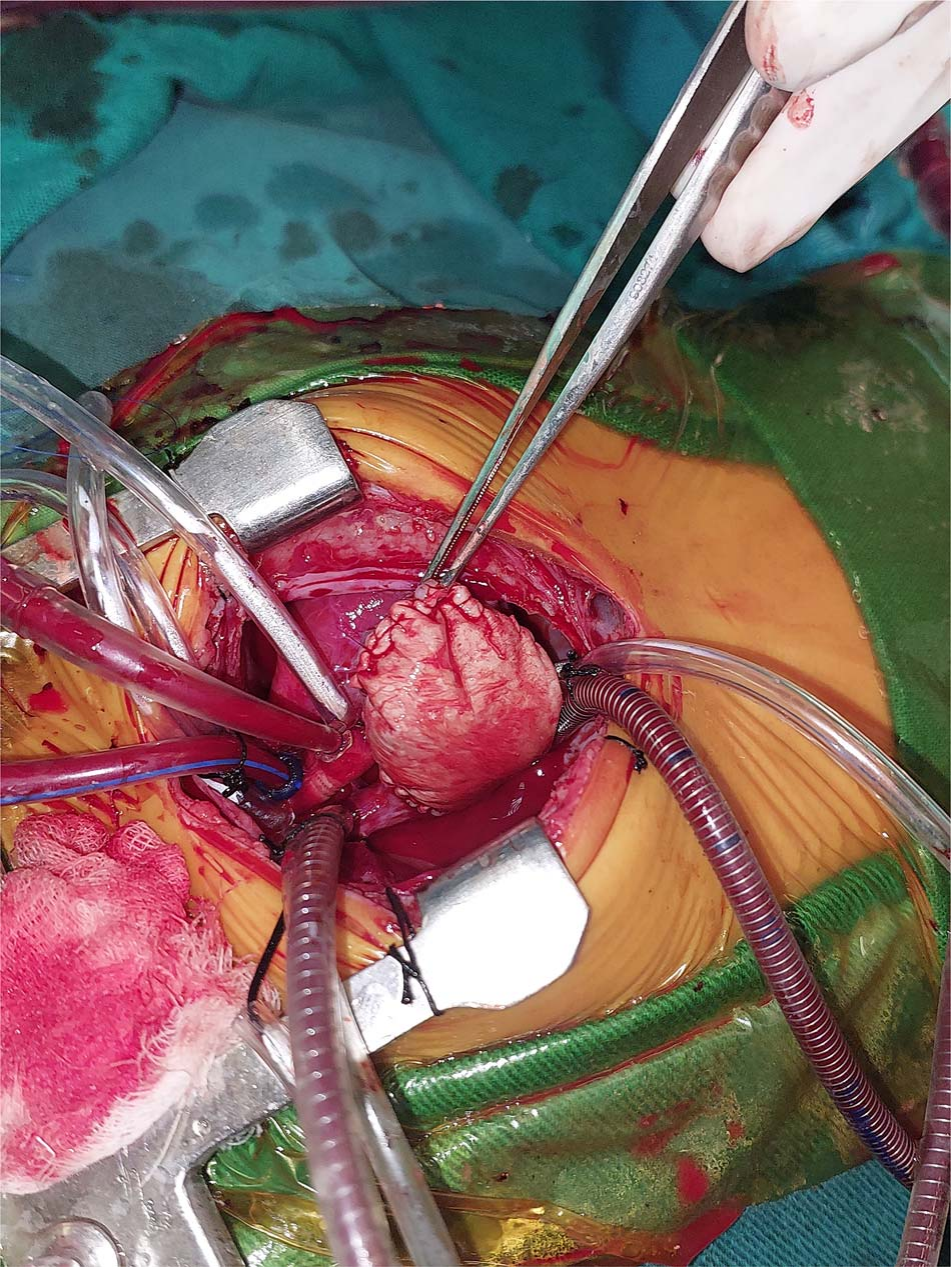
Intraoperative image showing the reconstructed right atrium with a bovine pericardial patch.

**Figure 7:**
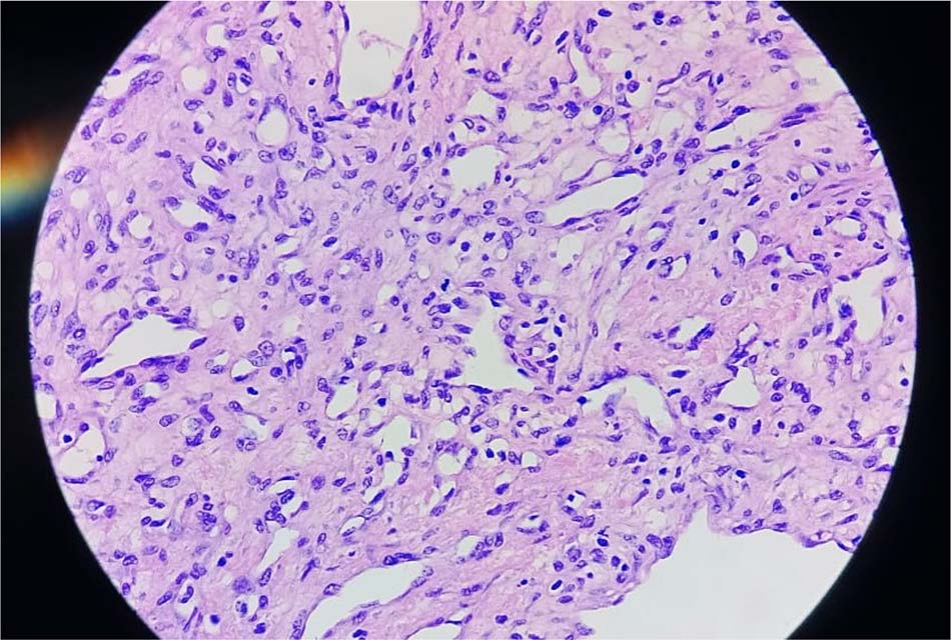
Histopathological image of the tumour.

A 2-month-old female infant was admitted with respiratory distress characterized by tachypnea, and cough, along with poor feeding and systemic symptoms such as fever and episodic diaphoresis. At 25 days of age, she had previously been hospitalized for similar symptoms, during which a massive pericardial effusion was identified. Initially, an extracardiac mass was suspected. Despite undergoing two pericardiocenteses and resuscitation after two post-cardiac arrest events, her clinical status continued to deteriorate, and she was referred for urgent surgical intervention due to cardiogenic shock from pericardial tamponade. On examination, she appeared pale with respiratory distress, intercostal retractions, pulsatile hepatomegaly (2 cm), splenomegaly (1 cm), and tachycardia (160 bpm). Laboratory findings showed hyponatremia (125 mmol/L). TTE demonstrated cardiomegaly, bilateral pleural effusion, and a massive pericardial effusion measuring 24–28 mm circumferentially. A 2.7 cm mass adjacent to the anterior RA wall and atrioventricular (AV) groove was identified (Fig. 1). Due to her unstable condition, CT imaging was deferred. An emergent median sternotomy revealed a severely distended pericardium containing copious effusion (Fig. 2). Upon opening the pericardium, immediate hemodynamic improvement was observed. The mass was visualized infiltrating the RA anterior wall and AV groove (Fig. 3). Total cardiopulmonary bypass (CPB) was initiated using aortic and bicaval cannulation. Following cardiac arrest with antegrade cold blood cardioplegia, the RA was incised posterior to the tumour. The lesion extended beyond the AV groove, precluding complete excision due to anatomical constraints (Fig. 4). Subtotal (debulking) resection was performed (Figs 4 and 5), and the RA anterior wall was reconstructed with a bovine pericardial patch (Fig. 6). The patient was successfully weaned from CPB, and the remainder of the surgery was uneventful. The infant was extubated within 48 hours and discharged after a 14-day hospital stay, with subsequent TTE showing satisfactory cardiac function. Histopathological evaluation confirmed a benign capillary hemangioma characterized by lobulated reddish tissue with compact capillary proliferation, fibrous septae, and stromal hyalinization. Immunohistochemical staining was positive for CD31 and CD34, confirming endothelial origin and ruling out malignancy (Fig. 7). At one-year follow-up, the patient remained in excellent general health with no signs of recurrence.

## Discussion

PCTs in infants, though rare, present significant diagnostic and therapeutic challenges, with an estimated incidence between 0.0017% and 0.28% [1]. While most pediatric PCTs are benign—commonly rhabdomyomas (60%)—other types include fibromas and hemangiomas [2, 3]. Malignant tumours are exceedingly rare in this age group [6].

Cardiac hemangiomas (CHs) account for only 2%–3% of all PCTs, with capillary hemangiomas being less common than other forms [4, 5]. They can occur in any chamber but show a predilection for the right atrium in neonates [7]. Although often asymptomatic, CHs can cause arrhythmias, heart failure, or outflow tract obstruction [8]. In neonates, non-specific symptoms like tachypnea and feeding difficulties often delay diagnosis [9].

Our patient presented at two months with respiratory distress, poor feeding, and a history of pericardial effusion and cardiac arrests, indicating severe hemodynamic compromise that necessitated urgent surgery. The tumour’s location on the anterior right atrium, extending into the atrioventricular (AV) groove, posed considerable surgical challenges due to its proximity to the conduction system and coronary vasculature. Due to the patient’s instability, preoperative imaging was limited to TTE. This is a common restriction in emergent neonatal cases where advanced modalities like MRI or CT are often precluded despite their superior anatomical delineation [10, 11].

Complete resection of CHs is often unachievable due to anatomical constraints. In our patient, tumour extension into the AV groove made complete removal impossible. Partial (debulking) resection was performed to restore hemodynamic stability, a valid approach supported in cases where tumour location precludes radical excision [12]. Histopathology confirmed a capillary hemangioma with immunohistochemical positivity for CD31 and CD34, confirming its vascular origin [13, 14].

Systemic therapies like corticosteroids and propranolol have limited application for cardiac tumours and are typically reserved for inoperable cases [15, 16]. The prognosis for pediatric CHs after surgery is generally favorable. Although recurrence can occur with incomplete resection, our patient showed sustained clinical improvement with no recurrence at one-year follow-up, consistent with other reports [17, 18]. Long-term echocardiographic surveillance remains essential.

## Conclusion

In conclusion, this case highlights a rare but severe manifestation of cardiac capillary hemangioma in early infancy, presenting with pericardial tamponade and cardiogenic shock. It underscores the importance of early recognition, rapid echocardiographic diagnosis, and timely surgical intervention. Our experience aligns with and expands upon the limited literature, reaffirming that partial resection can be both life-saving and sufficient to achieve long-term disease control in anatomically complex cases.

## Data Availability

The data that support the findings of this study are available from the corresponding author, upon reasonable request.

## Authors' declaration

None of the authors listed in the manuscript are employed by a government agency that has a primary function other than research and/or education. Moreover, none of the authors are submitting this manuscript as an official representative or on behalf of the government.
